# Causes of death and determinants of outcome in critically ill patients

**DOI:** 10.1186/cc5086

**Published:** 2006-11-03

**Authors:** Viktoria D Mayr, Martin W Dünser, Veronika Greil, Stefan Jochberger, Günter Luckner, Hanno Ulmer, Barbara E Friesenecker, Jukka Takala, Walter R Hasibeder

**Affiliations:** 1 Department of Anesthesiology and Critical Care Medicine, Innsbruck Medical University, Anichstrasse 35, 6020 Innsbruck, Austria; 2 Department of Intensive Care Medicine, University Hospital Bern, 3010 Bern, Switzerland; 3 Institute of Management and Quality Control, TILAK, Anichstrasse 35, 6020 Innsbruck, Austria; 4 Institute of Medical Biostatistics, Innsbruck Medical University, Anichstrasse 35, 6020 Innsbruck, Austria; 5 Department of Anesthesiology and Critical Care Medicine, Krankenhaus der Barmherzigen Schwestern, Ried im Innkreis, Austria

## Abstract

**Introduction:**

Whereas most studies focus on laboratory and clinical research, little is known about the causes of death and risk factors for death in critically ill patients.

**Methods:**

Three thousand seven hundred patients admitted to an adult intensive care unit (ICU) were prospectively evaluated. Study endpoints were to evaluate causes of death and risk factors for death in the ICU, in the hospital after discharge from ICU, and within one year after ICU admission. Causes of death in the ICU were defined according to standard ICU practice, whereas deaths in the hospital and at one year were defined and grouped according to the ICD-10 (International Statistical Classification of Diseases and Related Health Problems) score. Stepwise logistic regression analyses were separately calculated to identify independent risk factors for death during the given time periods.

**Results:**

Acute, refractory multiple organ dysfunction syndrome was the most frequent cause of death in the ICU (47%), and central nervous system failure (relative risk [RR] 16.07, 95% confidence interval [CI] 8.3 to 31.4, *p *< 0.001) and cardiovascular failure (RR 11.83, 95% CI 5.2 to 27.1, *p *< 0.001) were the two most important risk factors for death in the ICU. Malignant tumour disease and exacerbation of chronic cardiovascular disease were the most frequent causes of death in the hospital (31.3% and 19.4%, respectively) and at one year (33.2% and 16.1%, respectively).

**Conclusion:**

In this primarily surgical critically ill patient population, acute or chronic multiple organ dysfunction syndrome prevailed over single-organ failure or unexpected cardiac arrest as a cause of death in the ICU. Malignant tumour disease and chronic cardiovascular disease were the most important causes of death after ICU discharge.

## Introduction

In recent decades, intensive care medicine has developed into a highly specialised discipline covering several fields of medicine [1]. Whereas the total number of hospital beds in the United States decreased by 26.4% from 1985 to 2000, intensive care unit (ICU) beds increased by 26.2% during the same period [1], underlining the high demand for intensive care medicine. Mortality rates in the ICU strongly depend on the severity of illness and the patient population analysed. Across different ICUs, 6.4% to 40% of critically ill patients were reported to die despite intensive care medicine [2,3].

Although pathophysiological processes and new treatment approaches are extensively analysed in laboratory and clinical research, comparably less data are available on the causes of death, short- and long-term outcomes of critically ill patients, and associated risk factors. Mostly, data on specific prognostic criteria for single diseases have been published [4-11]. However, little is known of the exact causes of death and the impact of general risk factors that may uniformly complicate the course of critically ill patients irrespective of the underlying disease. Knowledge of such general determinants of outcome in a critically ill patient population would not only help improve prognostic evaluation of ICU patients, but also indicate what therapy and research should focus on to improve the short and long term outcomes of critically ill patients.

This prospective cohort study evaluates causes of death in a critically ill patient population in the ICU, in the hospital after ICU discharge, and within one year after ICU admission. Furthermore, independent risk factors for death during these periods are identified.

## Materials and methods

This prospective cohort study was conducted in a 12-bed general and surgical ICU in a tertiary, university teaching hospital with 1,595 beds. The ICU is one of six adult ICU facilities in the university hospital and primarily receives patients after elective or emergency surgery but also treats surgical and non-surgical patients with internal medical diseases. All patients admitted to this ICU between January 1, 1997, and December 31, 2003, were included in the study protocol. The study was approved by the institutional review board and the ethics committee of the Innsbruck Medical University (Innsbruck, Austria).

### Data collection and parameters

On admission to the ICU, pre-ICU data were documented in a standardised study protocol by the intensivist in charge. Pre-ICU data included the following: demographic variables (age and gender), admission diagnosis, type of admission (emergency or elective), referring unit (emergency department, operating theatre, recovery room, ward, or other ICU), type of disease (surgical or non-surgical), anatomical region of surgical intervention (cardiac, abdominal, traumatological, thoraco-abdominal, extremity, thoracic, neuro-, or spinal surgery), preoperative American Society of Anesthesiologic classification [12], specific data on cardiac surgery patients (preoperative ejection fraction, time on cardiopulmonary bypass, aortic cross-clamp time, and reperfusion time), history of pre-existent chronic diseases (chronic obstructive pulmonary disease, coronary heart disease, myocardial infarction within the preceding six months, myocardial infarction longer than six months before ICU admission, stable angina pectoris, unstable angina pectoris, congestive heart failure, treated chronic arterial hypertension, untreated chronic arterial hypertension, chronic renal insufficiency, chronic renal insufficiency requiring haemodialysis, liver cirrhosis, Child-Pugh classification of liver cirrhosis [13], insulin-dependent diabetes mellitus, non-insulin-dependent diabetes mellitus, healed tumour disease, malignant tumour disease, gastroduodenal ulcer disease, cerebrovascular insufficiency, status post-transient ischemic attack, prolonged ischemic neurological deficit or apoplectic insult, tetra- or paraplegia, other neurological pathology, psychiatric disease, immunosuppression, chemotherapy or radiation therapy during the preceding six months, chronic therapy with corticosteroids, and obesity), and the number of pre-existent chronic diseases. Presence or absence of pre-existent chronic diseases was documented in a binary fashion (0 = absent, 1 = present).

Any new complication or additional diagnosis that arose during the ICU stay was documented on a daily basis by one of three senior intensivists. Data included need for re-operation, massive transfusion, continuous veno-venous haemofiltration, or extracorporeal membrane oxygenation, as well as new-onset arrhythmias, SIRS (systemic inflammatory response syndrome), infection, sepsis, septic shock, acute respiratory distress syndrome, partial or global respiratory insufficiency, acute delirium, or critical illness polyneuropathy.

After discharge of the patient, data documentation was completed by one of the senior intensivists. Data documented at patient discharge included the Therapeutic Intervention Severity Score [14] and Simplified Acute Physiology Score (SAPS) II [15], which were both calculated from the worst physiological and laboratory parameters during the first 24 hours after ICU admission; highest multiple organ dysfunction syndrome (MODS) score (Appendix) during the ICU stay; worst PaO_2_/FiO_2 _ratio; creatinine, aspartate, alanine aminotransferase, and bilirubin serum concentrations during the ICU stay; duration of ICU stay in days; patient mortality; and type of unit the patient was transferred to (ward, cardiac surgical intermediate care unit, surgical intermediate care unit, other ICU, or other hospital). For all patients who died in the ICU, the cause of death was documented.

In January 2005, the demographic data of the study population were transferred to the Institute of Management and Quality Control of the university hospital, which documented the following data from all study patients: number of admissions to the ICU, hospital mortality, institution the patient was discharged to from hospital (home, other hospital, or rehabilitation unit), and causes of in-hospital death of critically ill patients after discharge from the ICU. At the same time, mortality data (death rate and cause of death according to the International Statistical Classification of Diseases and Related Health Problems [ICD-10] code [16]) were delivered by the 'Austrian Statistical Institution' as well as the 'Tumour Register' of South Tyrol. Using these data, mortality within one year after ICU admission and days of survival after ICU discharge were calculated for each study patient.

Before entry into the computer database, each case report was reviewed by a member of the study committee (senior intensivist or coworker). At the end of the electronic documentation of all study patients, plausibility tests were performed for each study variable to detect and correct typing mistakes that occurred during data entry or processing.

### Definitions and patient management

All codes and definitions of study variables were established before the beginning of the study and were uniformly documented as standard operating procedures for study data documentation. Study-related definitions are summarised in Table 1[16-21]. Cause of death was defined as the primary pathology (irrespective of its duration) leading to death of the patient or to the decision to withhold or withdraw intensive care therapy. Thus, cause of death did not necessarily have to be identical to admission diagnosis. To reduce inter-investigator variability to a minimum, all study-relevant decisions on cause of death, occurrence of new complications in the ICU, as well as any decision to withhold or withdraw intensive care treatment were made exclusively by one of three senior intensivists heading the ICU and in charge of the study.

#### Patient management

In all study patients, discharge from the ICU was initiated by senior intensivists only. According to institutional practice, cardiac surgery patients were routinely discharged to a cardiac surgical intermediate care unit. Only if no bed was available in this unit were cardiac surgery patients transferred directly from the ICU to the normal cardiac surgery ward. In all other patients, the decision to transfer the patient to other ICUs, intermediate care units, or normal wards was made on a patient-to-patient basis according to the condition and requirements of the patient.

#### Decision to withhold or withdraw life-sustaining therapy

The decision to withhold or withdraw life-sustaining therapy in a critically ill patient was made exclusively by two or more senior intensivists in agreement with the patient or the next-of-kin, as well as physicians from consulting departments other than the ICU. Aside from the extent of therapeutic support and the degree of organ dysfunction, decisions to withhold or withdraw life-sustaining therapy were based on the patient's will and, if the patient was not able to communicate, on the perceptions of the next-of-kin and physicians concerning the patient's preference about the use of ongoing life support, as well as predictions on the likelihood of survival in the ICU and future quality of life. Withdrawal of life-sustaining therapy in most cases included cessation of cardiovascular support and/or extracorporeal therapies (for example, continuous veno-venous haemofiltration or extracorporeal membrane oxygenation). All patients in whom life-sustaining therapy was withdrawn received intravenous benzodiazepines and opioids, fluid therapy, as well as mechanical ventilation, if necessary. In no patient was extubation or active termination of mechanical ventilation or tube feeding performed. Moreover, patients were not discharged to a ward when death was expected. Withholding of life-sustaining treatment included limitation of ongoing organ support (for example, limitation of the extent of cardiovascular support) or limitation of therapeutic support if organ failure occurred (for example, no continuous veno-venous haemofiltration if acute renal failure occurred). Thus, the decision to withhold life-sustaining treatments was also implemented in patients in whom the limiting organ failure had not yet been present.

### Outcome variables and study endpoints

The primary study endpoint was to evaluate the causes of death of critically ill patients in the ICU, in the hospital after discharge from the ICU, and within one year after admission to the ICU. The secondary study endpoint was to define risk factors for death in the ICU, in the hospital after discharge from the ICU, and within one year after admission to the ICU.

### Statistical analysis

Descriptive statistical methods were used to analyse demographic and clinical data of the study population, as well as causes of death. Three separate logistic regression analyses applying forward conditioning variables only were used to examine the association between study variables and ICU mortality (first analysis, denominator: death in the ICU), in-hospital mortality (second analysis, denominator: death in the hospital after discharge from the ICU), and mortality within one year after admission to the ICU (third analysis, denominator: death after hospital discharge and within one year after ICU admission). Variable selection for the three models was separately based on univariate comparisons. In each analysis, variables that were statistically significant at α = 0.05 in univariate comparisons were introduced into a multivariate model; covariates significant at <0.05 were retained in the model. To evaluate associations between single-organ functions and outcome variables, MODS score was not directly entered into the statistical model but was divided into its seven components (lungs, kidney, cardiovascular system, liver, coagulation, gastrointestinal tract, and central nervous system). According to the score (Appendix), these components were again subdivided into unaffected organ function (0 points), organ dysfunction (1 point), and organ failure (2 points). In both models, the MODS score was tested as contrasts of failure versus unaffected organ function, as well as organ dysfunction versus unaffected organ function. Tests for differences between study subgroups were performed using the unpaired Student *t*, χ^2 ^, or Mann-Whitney *U*-rank sum tests, as appropriate. Kaplan-Meier curves together with the log-rank sum test were used to illustrate cumulative survival rates for patients with and without central nervous system failure or cardiovascular failure in the ICU. A standard statistical program was used for all analyses of this study (SPSS 12.0 for Windows; SPSS Inc., Chicago, IL, USA). Data are given as mean values ± standard deviation unless stated otherwise.

## Results

### Study population and patient characteristics

**Figure 1:**
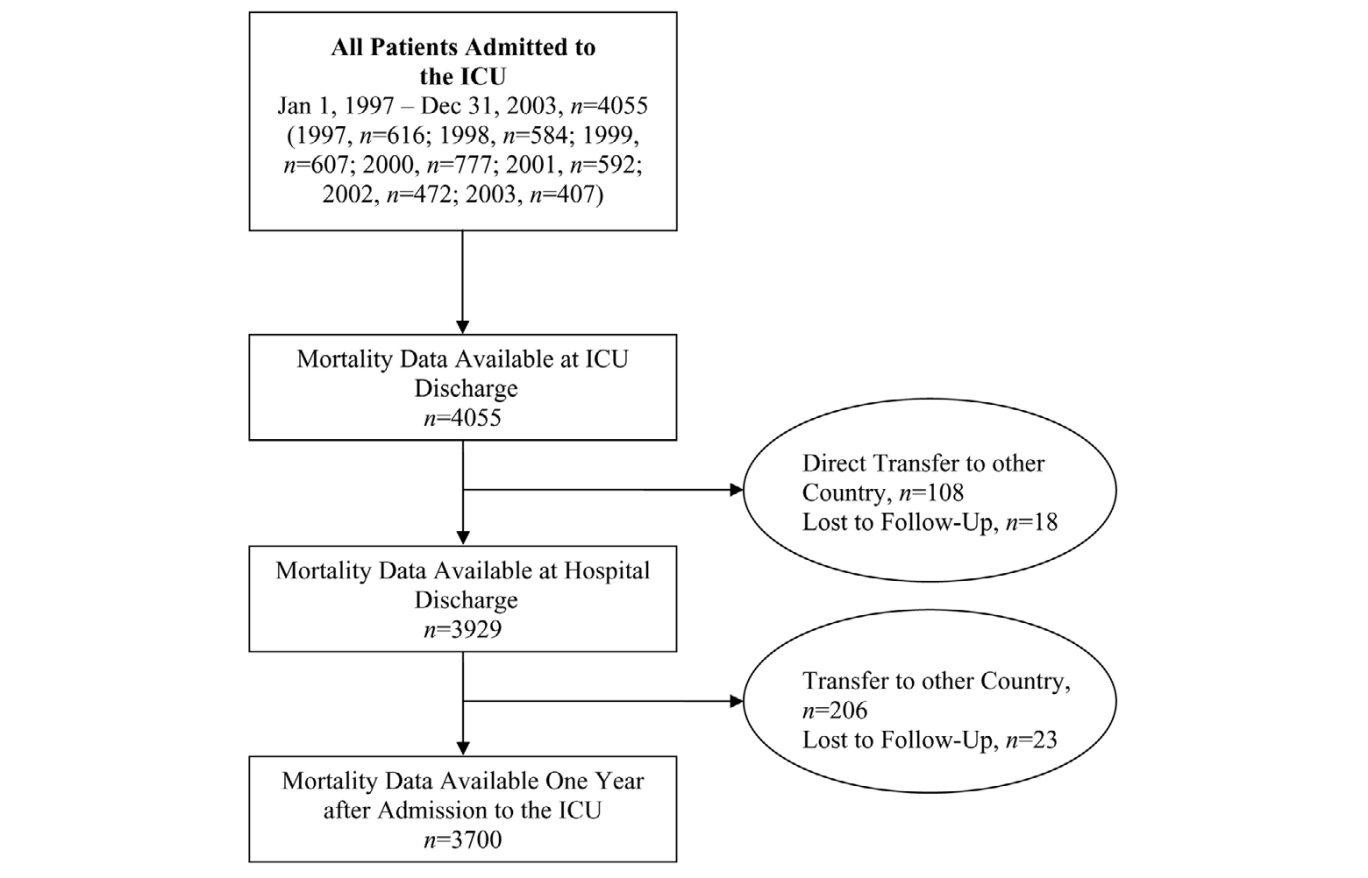
Overview of data inclusion. ICU, intensive care unit.

During the observation period, a total of 4,055 critically ill patients were admitted to the ICU, of whom 3,700 were included in the statistical analysis (Figure 1). Tables 2 to 4 give characteristics of the study population.

### ICU outcome

ICU mortality was 9.5% (353/3,700) for the study population for which full data sets were available one year after ICU admission and 8.7% (353/4,055) for all patients treated in the ICU during the given period. ICU mortality in patients admitted to the ICU because of infection, sepsis, or septic shock was 10.1%, 17.5%, and 53.3%, respectively.

**Figure 2:**
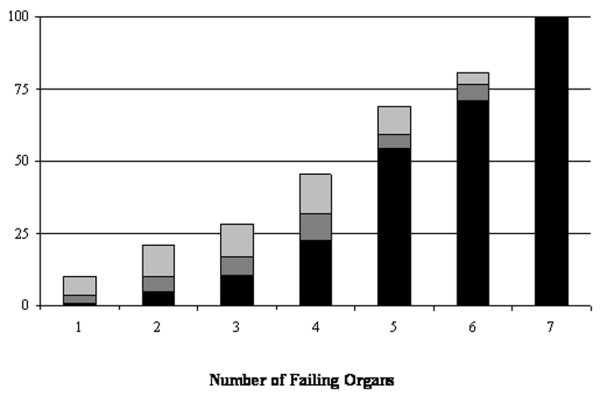
Mortality (percentage) of critically ill patients with different numbers of failing organs at intensive care unit discharge (black), hospital discharge (black + dark grey), and 1 year after intensive care unit admission (black + dark grey + light grey).

ICU survivors had a significantly shorter ICU stay than did non-survivors (7.6 ± 9.5 versus 11.7 ± 11.5 days, *p *< 0.001). No study patient died within the first day of ICU therapy. Twelve percent of non-survivors died within the first 3 days in the ICU, and 52.7% died within the first week after ICU admission. In end-of-life-decisions, treatment was withdrawn in 54.7% (193/353) of patients who died in the ICU. Table 5 summarises the causes of death of critically ill patients in the ICU. Acute, refractory MODS was the most frequent cause of death. Figure 2 presents the relationship between the number of failing organs and mortality at ICU discharge, hospital discharge, and one year after ICU admission. When the MODS score reached 14 points (failure of all seven evaluated organ systems) (*n *= 6), ICU mortality was 100%.

**Figure 3:**
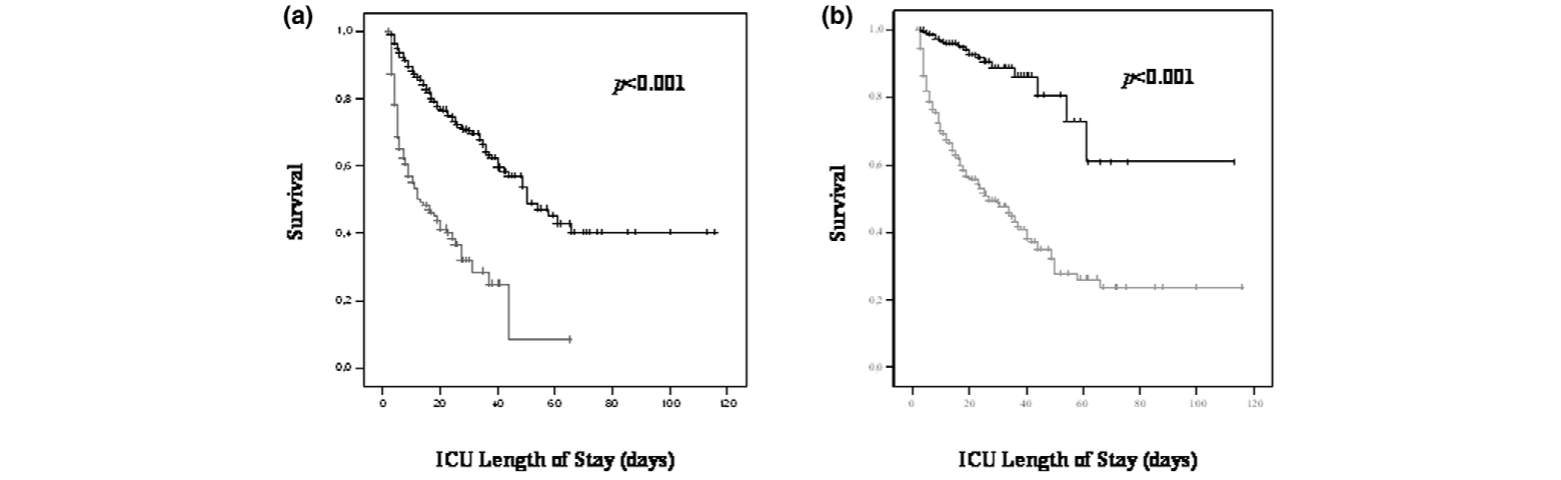
Independent risk factors for death in the ICU. Kaplan-Meier curves of critically ill patients with (grey) and without (black) central nervous system failure **(a) **and cardiovascular failure **(b)**. ICU, intensive care unit.

Independent risk factors for death in the ICU are shown in Table 6. Central nervous system failure and cardiovascular failure were the two most important risk factors for death in the ICU. Figure 3 displays Kaplan-Meier curves with the log-rank sum test for ICU patients with and without central nervous system failure (a) or cardiovascular failure (b). Patients with central nervous system or cardiovascular failure had a significantly higher ICU mortality rate than did patients without central nervous system failure (7.7% versus 54.2%, *p *< 0.001) or cardiovascular failure (1.4% versus 40.5%, *p *< 0.001). When compared with patients who were admitted from the operating theatre, emergency department, normal ward, or other ICUs, patients who were admitted to the ICU from the recovery room were older (61.9 ± 19 versus 59 ± 19 years, *p *= .002), had more pre-existent diseases (2.8 ± 1.9 versus 2.4 ± 1.6, *p *< 0.001), a higher American Society of Anesthesiologists classification (3.4 ± 0.9 versus 3.2 ± 0.9, *p *< 0.001), and a higher SAPS II (39.7 ± 17.9 versus 35.6 ± 14.8, *p *< 0.001).

### In-hospital outcome

In-hospital mortality after discharge from the ICU was 4.3% (144/3,347). Overall mortality of critically ill patients in the hospital was 13.5% (497/3,700). In-hospital mortality of patients admitted to the ICU because of infection, sepsis, or septic shock was 18.1%, 27.8%, and 57.2%, respectively. The mean duration of stay in the hospital after ICU discharge was significantly longer in patients who died in the hospital than in those who were discharged home or to another hospital (50.1 ± 62.8 versus 37.3 ± 53.3 days, *p *= 0.021); 101 patients discharged from the ICU (3%) had to be re-admitted to the ICU.

Table 5 summarises the most frequent causes of death of critically ill patients who died in the hospital after discharge from the ICU. Malignant tumour disease was the most frequent cause of in-hospital death of critically ill patients after discharge from the ICU. Table 6 presents independent risk factors for death of critically ill patients in the hospital.

#### 

### One year outcome

After discharge from the hospital, mortality within one year after admission to the ICU was 8.9% (286/3,203). Overall mortality of critically ill patients within one year after admission to the ICU was 21.2% (783/3,700). One-year mortality of patients admitted to the ICU because of infection, sepsis, or septic shock was 33.6%, 42.3%, and 66.9%, respectively.

In patients who died within one year after admission to the ICU, the mean time after discharge from the hospital to death was 133 ± 108 days. Table 5 displays the most frequent causes of death of critically ill patients who died within one year after ICU admission. Tumour disease was the most frequent cause of death. Fifty-five percent of the non-survivors died from the same condition that prompted admission to the ICU.

Table 6 shows independent risk factors for death of critically ill patients within one year after admission to the ICU. The number of ICU admissions was the most important risk factor for death. Patients who were treated more than once in the ICU were significantly more likely to die within the first year after ICU admission than patients who required only one admission to the ICU (30.7% versus 21%, *p *= 0.026).

## Discussion

When taking the high degree of physiologic derangement (SAPS II, 37.6 ± 16 points) and the associated predicted mortality (19.7%) into account, the observed ICU mortality of 9.5% in this patient population is low. An important explanation for this observation may be the high proportion of postoperative patients, in particular postoperative cardiac surgery patients, in this study population. In contrast to earlier reports [22,23], ICU non-survivors did not die early in the course of the disease but primarily in the period of prolonged critical illness (11.7 ± 11.5 days). This finding is in accordance with recent studies [24] and underlines the emerging phenomenon of chronic critical illness [21,25]. The rate of withdrawal of life-sustaining therapy in this cohort study is in accordance with a recent multicentre trial [23] and with findings that decisions to forgo cardiopulmonary resuscitation precede 60% to 90% of deaths in the ICU [26,27].

Whereas acute MODS (*n *= 166/353, 47%) was the most important cause of death in the ICU, only very few patients died of single-organ failure (*n *= 44/353, 12.3%). In 41/353 patients (11.6%), inability to fully recover from acute critical illness led to chronic MODS and death. The important role of MODS, particularly its chronic form, is further confirmed by the results of major studies [24,28]. Unexpected cardiac arrest was only a rare cause of death in the ICU.

The two most important risk factors for death in the ICU were presence of either central nervous system failure or cardiovascular failure. Although central nervous system failure increased the risk of death most significantly (relative risk [RR] 16.07, 95% confidence interval [CI] 8.2 to 31.4), only a comparably small number of patients suffered from it (144/3,700, 3.9%; primarily severe brain trauma or post-cardiopulmonary resuscitation). On the other hand, cardiovascular failure occurred in 20.2% (747/3,700) of ICU patients, increasing the risk of death 11.8-fold. Impaired organ perfusion has been suggested as a contributing factor in the development of organ dysfunction [29]. Recent data underline the strong prognostic impact of hypotension and cardiovascular failure in critically ill patients with sepsis [30,31]. Although acute renal failure as a single-organ failure or part of the MODS had a highly significant impact on ICU survival in several previous studies [7,32,33], acute renal failure requiring renal replacement therapy only moderately increased the risk of death in this study population.

In contrast to published data on in-hospital mortality of critically ill patients after ICU discharge (6.1% to 35.4%) [34-36], the observed in-hospital mortality of 4.9% after discharge from the ICU is comparably low. Given that this was an uncontrolled study, the reason for this finding cannot be extracted from the data. However, it can be speculated that the high percentage of patients (65.4%) discharged from the ICU to intermediate care units with facilities for continuous monitoring of vital organ functions may have contributed to the low in-hospital mortality. This hypothesis is supported by the results of other authors [37-39]. Additionally, the in-hospital mortality rate may appear low because 48.8% of the patients discharged from the hospital were transferred either to another hospital or to a rehabilitation facility.

Malignant tumour disease was the most frequent cause of death of critically ill patients in the hospital after ICU discharge. Obviously, these patients survived the period of critical illness with *only *a moderate increase in additional mortality (RR 2.19, 95% CI 1.4 to 3.4). However, relapsing impairment of vital organ functions and subsequent death occurred in a large portion of these patients after discharge from the ICU (45/459, 9.8%). In view of the malignant character of the underlying disease, clinicians may have refrained from re-admission to the ICU [2,40]. Exacerbation of chronic cardiovascular disease and liver disease were, respectively, the second and third most frequent causes of in-hospital death of critically ill patients after ICU discharge. Similarly, it is conceivable that critical illness put too high a strain on chronically dysfunctional organs, which could be temporarily compensated by ICU therapy but later decompensated. The high incidence of malignant tumour disease among patients who underwent major abdominal surgery (250/674, 37.1%) and the high incidence of central nervous system failure in neurosurgical patients (14/125, 11.2%) may partially explain why, among other factors, abdominal surgery and neurosurgery were important risk factors for in-hospital death of critically ill patients after ICU discharge.

Mortality at one year after ICU admission in this study population (21.2%) seems particularly low for patients with more than four failing organs (Figure 2). This finding is even more astonishing given the comparably large number of patients in these high-organ-failure groups (four failing organs, *n *= 258; five failing organs, *n *= 234; six failing organs, *n *= 121) and the mean age within the distribution of the study population (four failing organs, 64 ± 16 years; five failing organs, 66 ± 14 years; six failing organs, 65 ± 14 years). Similar to the causes of in-hospital death, malignant tumour disease and cardiovascular disease were the most frequent causes of death of critically ill patients one year after ICU admission. This finding is in agreement with the results of an earlier study by Ridley *et al. *[41], who identified malignancy and respiratory failure as the two most common causes of death of survivors of critical illness. An explanation for the high incidence of malignant tumour disease as a cause of death of critically ill patients after hospital discharge may be significant critical illness-associated immune suppression, which is known to have permissive effects on tumour growth and progression [42].

Need for re-admission to the ICU was by far the most important risk factor for death. However, it cannot be determined from the results of this study whether increased mortality resulted from re-admission itself or was simply an epiphenomenon of the severe underlying disease. In agreement with the results of other studies, it can be hypothesised that prevention of ICU re-admission could have significantly improved long-term outcome of these critically ill patients [43,44]. Although acute renal failure played only a comparably minor role for ICU mortality as compared with central nervous system or cardiovascular failure, it was the only type of organ failure that had a significant impact on long-term survival of critically ill patients. Patients who sustained acute renal failure in the ICU died primarily from diseases other than chronic renal pathologies. The importance of renal function for the long-term prognosis of critically ill patients has already been suggested by earlier studies [45].

Even though the number of pre-existent diseases correlated with a significantly longer stay in the ICU (*p *= 0.001) and the hospital (*p *= 0.001), it had no influence on patient outcome during this period. However, the number of pre-existent diseases significantly reduced one year survival in this critically ill patient population. This observation can be explained simply by progression of the underlying disease but might also be associated with a more rapid progression of chronic disease after the period of critical illness with a high degree of physiologic derangement (SAPS II).

When interpreting the results of this study, important limitations need to be considered. First, this cohort study was conducted as a single-centre study. Although this yielded a therapeutically homogeneous study population, it precludes wide generalisation of our results to other centres because of institution-based differences in treatment, patient population, and admission policies. Particularly in view of its high proportion of postoperative critically ill patients, this study population may not be comparable with critically ill medical or neurology patients. Second, in contrast to the more widely used SOFA (Sequential Organ Failure Assessment) score, the MODS score used in this study differentiates only between organ dysfunction (1 point) and organ failure (2 points). This might have overestimated the number of failing organs and thus survival rates. Third, causes of death given in this study do not necessarily reflect mechanisms of death. Furthermore, ICD classification has been considered to be inaccurate in some countries because it is largely based on death certificates, which are often completed by clinicians who were not familiar with the patients. Even though early reports on the reliability and validity of ICD-10 documentation in the German-speaking countries (Germany, Austria, and Switzerland) yielded favourable results [46], it cannot be ruled out that this limitation might have influenced the reliability of our data on causes of death after discharge from the ICU. Fourth, although numerous variables have been introduced to the statistical analysis, important parameters may have been omitted and their impact on patient outcome thus missed. For example, genetic variables are not routinely measured but have been reported to be of significant importance for the outcome of critically ill patients [47]. Fifth, pre-existent diseases were documented in a binary fashion only and therefore did not consider the severity of chronic diseases. This might have limited interpretation of the impact of severe degrees of chronic illness.

## Conclusion

In this primarily surgical critically ill patient population, acute or chronic MODS prevails by far over single-organ failure or unexpected cardiac arrest as cause of death in the ICU. Malignant tumour disease and exacerbation of chronic cardiovascular disease were the most frequent causes of death of critically ill patients in the hospital after ICU discharge and 1 year after ICU admission. To improve short- and long-term outcomes of critically ill patients, treatment and research should focus on effective therapy of central nervous system failure and cardiovascular failure, as well as on prevention of re-admission to the ICU.

## Key messages

• Acute, refractory MODS was the most frequent cause of death in the ICU.

• Central nervous system failure and cardiovascular failure were the two most important risk factors for death in the ICU.

• Malignant tumour disease and exacerbation of chronic cardiovascular disease were the most frequent causes of death in the hospital and at one year.

## Abbreviations

CI = confidence interval; ICD-10 = International Statistical Classification of Diseases and Related Health Problems; ICU = intensive care unit; MODS = multiple organ dysfunction syndrome; RR = relative risk; SAPS = Simplified Acute Physiology Score.

## Competing interests

The authors declare that they have no competing interests.

## Authors' contributions

VM conceived the study protocol, participated in its design and coordination, carried out the experiment and documentation, and drafted the manuscript. MD conceived the study protocol, participated in the study design and its coordination, carried out the experiment and documentation, helped perform statistical analysis, and drafted the manuscript. VG performed the data documentation. SJ carried out the experiment and documentation. GL carried out the experiment and documentation. HU performed statistical analysis and helped interpret the data. BF participated in coordinating the study, carried out the experiment and documentation, and helped draft the manuscript. JT critically revised the manuscript for important intellectual content. WH conceived the study protocol, participated in the study design and its coordination, carried out the experiment and documentation, helped interpret the data, and critically revised the manuscript for important intellectual content. All authors read and approved the final version of the manuscript.

## Appendix

See table 7, modified from [48].



## Figures and Tables

**Table 1:** Study definitions

Obesity	Body mass index >30 kg/m^2 ^[17]
Massive transfusion	Replacement of one blood mass within 24 hours or need for transfusion of four red cell concentrates within one hour [18]
SIRS, sepsis, and septic shock	Definitions according to standard recommendations [19]
ARDS	Acute deterioration of gas exchange (PaO_2_/FiO_2 _ratio <200), bilateral infiltrates on the chest x-ray, pulmonary capillary wedge pressure <18 mmHg [20]
Partial respiratory insufficiency	PaO_2 _<60 mmHg in the extubated spontaneously breathing patient with or without oxygen
Global respiratory insufficiency	PaO_2 _<60 mmHg and PaCO_2 _>60 mmHg in the extubated spontaneously breathing patient with or without oxygen
Causes of death^a ^	
Cardiovascular failure	According to the MODS score given in the Appendix
Irreversible cardiovascular failure	Death in pharmacologically uncontrollable hypotension (MAP <60 mmHg)
Acute, refractory MODS	Death from severe MODS (>four failing organs), MAP >60 mmHg, metabolic derangement (for example, lactic acidosis with arterial lactate concentrations >100 mg/dl)
Chronic, refractory MODS	Death from a secondary complication leading to MODS in the state of chronic critical illness
Chronic critical illness	Period after tracheotomy has been performed on the ICU because of long-term ventilation (>7 to 12 days) [21]

^a ^Any other cause of death in the ICU, in the hospital after discharge from the ICU, and within one year after admission to the ICU was defined and grouped according to the ICD-10 code [16]. ARDS, acute respiratory distress syndrome; FiO_2_, inspiratory oxygen concentration; ICD-10, International Statistical Classification of Diseases and Related Health Problems; ICU, intensive care unit; MAP, mean arterial blood pressure; MODS, multiple organ dysfunction syndrome; PaCO_2_, partial arterial carbon dioxide pressure; PaO_2_, partial arterial oxygen pressure; SIRS, systemic inflammatory response syndrome.

**Table 2:** Characteristics of study patients (*n *= 3,700)

Characteristic	*n *(percentage)^a ^
Male gender	2,390 (64.6)
Age	59.2 ± 19.3 years
>65 years	1,702 (46)
>75 years	584 (15.8)
Obesity	466 (12.6)
Emergency admission	1,322 (35.7)
Surgical disease	2,950 (79.7)
ASA classification	3.3 ± 0.9
Number of pre-existent diseases	2.4 ± 1.7
Referral unit	
Operation theatre	2,717 (73.5)
Recovery room	454 (12.3)
Emergency department	2,950 (79.7)
Normal ward	140 (3.8)
Other ICU	69 (1.9)
Reason for ICU admission	
Post-cardiac surgery	45.4%
Multiple trauma	7.1%
Major tumour surgery	4.7%
Abdominal sepsis	4.5%
Acute abdominal disease other than sepsis	3.6%
Respiratory insufficiency	3.4%
ICU duration of stay	8 ± 9.8 days
>7 days	962 (26)
>14 days	491 (13.3)
>21 days	281 (7.6)

^a ^Except where other units are given. Data are given as mean values ± standard deviation except where indicated otherwise. ASA, American Society of Anesthesiologists; ICU, intensive care unit.

**Table 3:** Characteristics of study patients during intensive care unit stay

Characteristic	*n *(percentage)^a ^
SAPS II	37.6 ± 16 points
TISS-28	49.8 ± 10 points
MODS score	2.8 ± 3.9 points
Organ failures	
Lung failure	1,225 (33.1)
Liver failure	772 (20.9)
Cardiovascular failure	771 (20.8)
Coagulation failure	492 (13.3)
Renal failure	486 (13.1)
Central nervous system failure	144 (3.9)
Gastrointestinal failure	95 (2.6)
Need for re-operation	217 (5.9)
Massive transfusion	635 (17.2)
New-onset arrhythmias	877 (23.7)
ARDS	669 (18.1)
Partial respiratory insufficiency	2,045 (55.3)
Global respiratory insufficiency	124 (3.4)
SIRS	690 (18.7)
Infection	367 (9.9)
Sepsis	268 (7.2)
Septic shock	276 (7.5)
Acute delirium	754 (20.4)
Critical illness polyneuropathy	233 (6.3)
Continuous veno-venous haemofiltration	494 (13.4)
Extracoporeal membrane oxygenation	31 (0.8)

^a ^Except where other units are given. Data are given as mean values ± standard deviation except where indicated otherwise. ARDS, Acute Respiratory Distress Syndrome; MODS, multiple organ dysfunction syndrome; SAPS, Simplified Acute Physiology Score; SIRS, systemic inflammatory response syndrome; TISS, Therapeutic Intervention Severity Score.

**Table 4:** Characteristics of study patients after ICU Stay

Characteristic	*n *(percentage)^a ^
ICU discharge unit	
Normal ward	872 (23.6)
Cardiac surgical interCU	1,674 (45.2)
Surgical interCU	746 (20.2)
Other ICU	41 (1.1)
Transfer to other hospital	33 (0.9)
Hospital duration of stay	37.9 ± 53.9 days
ICU re-admission	101 (3)
Hospital discharge	
Home	1,632 (51.2)
Other hospital/rehab	1,554 (48.8)

^a ^Except where other units are given. Data are given as mean values ± standard deviation except where indicated otherwise. ICU, intensive care unit; interCU, intermediate care unit; rehab, rehabilitation unit.

**Table 5:** Causes of death of critically ill patients

	Percentage	*n*
Causes of death in the intensive care unit (ICU)		
Acute, refractory multiple organ dysfunction syndrome	47	166/353
Refractory cardiovascular failure	17.8	63/353
Refractory, chronic multiple organ dysfunction syndrome	11.6	41/353
Central nervous system failure	7.9	28/353
Acute cardiac arrest	4.2	15/353
End-stage tumour disease	3.4	12/353
Acute haemorrhage	3.4	12/353
Intractable intestinal ischemia	2.5	9/353
Pulmonary failure	1.1	4/353
Acute or chronic liver failure	0.8	3/353
Causes of death in the hospital		
Malignant tumour disease	31.3	45/144
Exacerbation of chronic cardiovascular disease	19.4	28/144
Exacerbation of chronic liver disease	11.8	17/144
Acute or chronic abdominal disease	6.9	10/144
Acute cerebral infarction	4.2	6/144
Acute or chronic renal disease	4.2	6/144
Infectious disease	3.5	5/144
Acute cardiac event/cardiac arrest	3.5	5/144
Others	15.3	22/144
Causes of death within 1 year after ICU admission		
Malignant tumour disease	33.2	95/286
Exacerbation of chronic cardiovascular disease	16.1	46/286
Acute cardiovascular disease	7.7	22/286
Cerebral infarction	4.2	12/286
Chronic renal disease	2.4	7/286
Diseases of the gastrointestinal tract	2.1	6/286
Acute or chronic liver disease	1.7	5/286
Diseases of the blood or immune system	1.4	4/286
Trauma	1.4	4/286
Chronic pulmonary disease	1	3/286
Infectious diseases	1	3/286
Thrombotic diseases	1	3/286
Neurologic diseases	0.7	2/286
Acute bleeding	0.7	2/286
Suicide	0.7	2/286
Others	24.5	70/286

**Table 6:** Independent risk factors for death of critically ill patients

	Relative risk	95% CI	*p *value
Death in the ICU			
Central nervous system failure	16.07	8.2 to 31.4	<0.001
Cardiovascular failure	11.83	5.2 to 27.1	<0.001
Acute renal failure	2.7	1.7 to 4.3	<0.001
Admission from recovery room	2.2	1.4 to 3.4	<0.001
Malignant tumour disease	2.19	1.4 to 3.4	<0.001
Death in the hospital			
Neurosurgery	6.75	3.3 to 13.8	0.015
Non-surgical, internal disease	6.75	3.3 to 13.8	<0.001
Abdominal surgery	5.09	3.2 to 8.2	<0.001
Central nervous system failure	5.08	2.2 to 11.6	<0.001
Cardiovascular failure	3.85	2.1 to 6.9	<0.001
Death within one year after ICU admission			
Number of admissions to the ICU	11.84	4.3 to 32.5	<0.001
Acute renal failure	2.97	1.2 to 7.6	0.024
Number of pre-existent diseases	1.41	1.1 to 1.7	0.001
SAPS II (per unit increase)	1.12	1.1 to 1.2	<0.001

CI, confidence interval; ICU, intensive care unit; SAPS, Simplified Acute Physiology Score.

**Table 7:** Definitions and grading of organ dysfunction (MODS score)

Function	No organ dysfunction/failure	Organ dysfunction	Organ failure
Pulmonary	PaO_2_/FiO_2 _ratio ≥ 300	PaO_2_/FiO_2 _ratio ≥ 250	PaO_2_/FiO_2 _ratio <250
Renal	Creatinine ≤ 2.0 mg/dl	Creatinine >2.0 mg/dl; doubling of creatinine in patients with previous compensated renal failure	Continuous veno-venous haemofiltration
Hepatic	Bilirubin <2 mg/dl; ASAT/ALAT within normal range	Bilirubin 2 to 5 mg/dl; ASAT/ALAT ≤ three times normal value	Bilirubin >5 mg/dl; ASAT/ALAT > three times normal value
Haematologic	Thrombocytes within normal range; normal coagulation	Thrombocyte decrease ≥ 25%; abnormal PT/aPTT with and without bleeding	Haemorrhagic diathesis; massive transfusion five blood products per hour or > 10 blood products per 24 hours
Cardiovascular	Normal blood pressure; no vasoactive drugs except dopamine ≤ 5 μg/kg per minute	Fluid resuscitation > 50% of normal need and/or dopamine >5 μg/kg per minute, dobutamine <10 μg/kg per minute, phenylephrine	Dobutamine >10 μg/kg per minute, AVP, epinephrine, norepinephrine, combination of catecholamines, IABP, VAD
Gastrointestinal	Normal gastrointestinal function, no bleeding	Ileus >7 days or bleeding requiring ≤ six blood products per 24 hours	Massive bleeding requiring > six blood products per 24 hours
Central nervous system	GCS ≥ 12	GCS 9–11	GCS ≤ 8

Modified from [48]. ALAT, alanin-aminotransferase; aPTT, activated thromboplastin time; ASAT, aspartat-aminotransferase; AVP, arginine vasopressin; FiO_2_, fractional inspiratory oxygen concentration; GCS, Glasgow Coma Scale; IABP, intra-aortic balloon pump; MODS, multiple organ dysfunction syndrome; PaO_2_, arterial oxygen tension; PT, prothrombin time; VAD, ventricular assist device.

